# Transplantation impacts on the oral microbiome of kidney recipients and donors

**DOI:** 10.3389/frmbi.2023.1258290

**Published:** 2023-11-03

**Authors:** Paul M. Campbell, Thomas Willmott, Gavin J. Humphreys, Oana Piscoran, Houda Chea, Angela M. Summers, Joanne E. Konkel, Christopher G. Knight, Titus Augustine, Andrew J. McBain

**Affiliations:** ^1^ School of Health Sciences, Faculty of Biology, Medicine and Health, The University of Manchester, Manchester, United Kingdom; ^2^ Manchester Centre for Transplantation, Manchester Royal Infirmary, Manchester University NHS Foundation Trust, Manchester Academic Health Science Centre, Manchester, United Kingdom; ^3^ Lydia Becker Institute of Immunology and Inflammation, Faculty of Biology, Medicine and Health, The University of Manchester, Manchester, United Kingdom; ^4^ School of Natural Sciences, Faculty of Science and Engineering, The University of Manchester, Manchester, United Kingdom

**Keywords:** oral microbiome, kidney transplant, immunosuppression, surgery, renal allograft, chronic kidney disease, end-stage renal disease, infective endocarditis

## Abstract

**Introduction:**

Chronic kidney disease (CKD) may affect the human microbiome via increased concentrations of uremic toxins such as urea and creatinine.

**Methods:**

We have profiled the oral microbiota in patients with CKD before and one week after kidney transplantation. Living kidney donors were also longitudinally tracked over a similar period, allowing direct comparison between a group undergoing transplant surgery alone (donors) (n=13) and a group additionally undergoing the introduction of immunosuppressive agents and the resolution of CKD (recipients) (n=45).

**Results:**

Transplantation was associated with a similar pattern of decreasing alpha diversity in the oral microbiome in recipients and donors via Kruskal-Wallis testing, within one week of transplantation. Amplicon sequence variants (ASVs) associated with *Haemophilus parainfluenzae*, *Aggregatibacteria segnis*, *Peptostreptococcus* and *Actinobacillu*s were significantly decreased in recipients within a week of transplantation.

**Discussion:**

A reduction in ASVs in these genera could influence the risk of bacterial endocarditis, a rare but high-mortality kidney transplantation complication. A range of factors may drive the observed changes in oral microbiome including both factors associated with surgery itself and the decreases in salivary urea, administration of macrolide antibiotic immunosuppressants, and disruption to immune function that characterise kidney transplant.

## 1 Introduction

Infection is the leading cause of morbidity and mortality following kidney transplantation, and infection occurs in 31% of recipients within two years (Karuthu and Blumberg, 2012; Cowan et al., 2018). It has been postulated that infections occurring within the first month of surgery could be derived from the hospital, the procedure, or from the donor (Karuthu and Blumberg, 2012). Infections occurring beyond the first month, but before six months, may be latent (relapsed, residual, or opportunistic) infections, where immunosuppression inhibits host defenses. Beyond 6 months, Karuthu and Blumberg (2012) have proposed that infections are likely to be community-acquired. With this paradigm of infection in mind, the increases in potentially opportunistic pathogens observed within the first month by Fricke et al. (2014) highlight the potential for the oral cavity to become a reservoir for infection elsewhere in the body. Extra-oral infections and inflammation can potentially cause or complicate cardiovascular disease, adverse pregnancy outcomes, rheumatoid arthritis, inflammatory bowel disease and colorectal cancer (Han and Wang, 2013). Inflammation is also a major regulator of drug-metabolizing enzymes and could affect immunosuppressive drug levels and interactions (Stanke-Labesque et al., 2020).

During kidney transplantation, prophylactic antimicrobial regimens and immunosuppression are administered to reduce the risk of opportunistic infection or allograft rejection, respectively (Fricke et al., 2014). Data from the ELITE-Symphony trial indicates these complications still occur in approximately 25% of cases within one year post-transplant (Cippà et al., 2015). Whilst immunosuppression and prophylactic antibiotics remain critical in post-operative care, they may affect the protective and beneficial functions of host microbiota.

Increased oral concentrations of toxins such as urea and creatinine have been reported in chronic kidney disease (CKD), and concentrations of urea are monitored during dialysis (Araújo et al., 2015; Lasisi et al., 2016; Simões-Silva et al., 2018; Campbell et al., 2020). Changes in the composition of the oral microbiota following kidney transplantation have been associated with oral lesions (Sahebjamee et al., 2010; Da Silva et al., 2012; Levarda-Hudolin et al., 2016) and with squamous oral carcinoma and other oral malignancies, although in both cases the causal link remains unclear (Regev et al., 1992; Thomas et al., 1993; Yoon et al., 2003). Renal transplant patients treated with immunosuppressants cyclosporine A or tacrolimus commonly suffer persistent gingival microbial overgrowth (Chu et al., 2000). A complete understanding of how kidney transplantation alters the oral microbiome and what downstream effects are elicited is however still required.

Culture-based studies have reported that total viable counts of microbes isolated from saliva increased 90 days following kidney transplantation, with an associated greater risk of gingival overgrowth (Saraiva et al., 2006). More recently, high-throughput sequencing methods have been employed to compare kidney transplant recipients with healthy controls. Whilst alpha and beta diversity were not found to be different for recipients (>1-year post-transplant), increased relative abundance of operational taxonomic units (OTUs) of potential opportunistic pathogens including *Klebsiella pneumoniae*, *Pseudomonas fluorescens*, *Pseudomonas aeruginosa*, *Acinetobacter* spp., *Vibrio* spp., and other *Enterobacteriaceae* spp. was observed (Diaz et al., 2013). Compared with a longitudinal study by Fricke et al. (2014) observing kidney transplant recipients who reported no change in oral microbiome diversity between any timepoint (before, 1 or 6 months post-transplant) decreases in presumed commensals *Aggregatibacter*, *Eikenella*, *Haemophilus*, *Leptotrichia*, *Neisseria*, *Peptostreptococcus* and *Tannerella* (between pre and 1-month post-transplant) were reported along with a similar increase in potential opportunistic pathogens: *Comamonas*, *Peptostreptococcus*, *Thiobacillus*, *Treponema* and *Veillonella* (between 1-month and 6-months post-transplant).

Organ transplantation and subsequent immunosuppression offer the opportunity to study the effects of immunosuppression longitudinally. However, comorbidities and other confounders make the task of isolating effects solely due to the suppression of the immune system difficult. Immunosuppressant drugs, such as tacrolimus, is a macrolide with antibiotic properties and is therefore likely to affect bacteria. Moreover, prophylactic antibiotics, frequently used in transplant patients, (Orlando et al., 2015; Bliven et al., 2018), may also significantly alter the microbiome (Jakobsson et al., 2010; Korpela et al., 2016; Campbell et al., 2020). The oral microbiome has vital functions in health maintenance and disease protection. We have profiled the salivary microbiomes of donors and recipients during the immediate period before and after surgery using high throughput 16S rRNA sequencing. This study aimed to gain further insight into changes in the oral microbiome of kidney transplant recipients and its consequences.

## 2 Methods

### 2.1 Study recruitment, ethics and design

Patients recruited were adults (aged between 18-75), able to give informed consent and were admitted to Manchester Royal Infirmary (MRI) in Manchester (United Kingdom) for renal transplant surgery as either a living donor or recipient. Sample collection was performed with approval from the Health Research Authority (HRA) and study protocol and documentation were given favourable ethical approval from the local research ethics committee (REC reference: 19/NW/0100). Saliva samples were collected from kidney transplant recipients at three time points: Timepoint A (Pre-Transplant) the day before the surgery, Timepoint B (Day-1 post-transplant) the day after the surgery and Timepoint C (Week-1 post-Transplant) on the first clinic visit post-surgery (often Day 7). For the living donors, saliva samples were collected at Timepoints A and B, however, Timepoint C (Week-1) sample was collected before discharge from the hospital (often Days 3-5). Saliva samples were collected by asking participants to spit 1ml of saliva into 2ml Eppendorf tubes (Eppendorf Ltd., U.K). Saliva samples were immediately stored at -80°C for later DNA extraction.

#### 2.1.1 16S rRNA gene amplification and sequencing

For recipients, 45 saliva samples were collected (from 45 volunteers) at Timepoint A, all 45 recipients were sampled at Timepoint B and 43 were sampled at Timepoint C. For donors, 13 samples (from 13 volunteers) were collected at Timepoint A, with all 13 re-sampled at Timepoint B and 10 sampled again at Timepoint C (169 samples in total). DNA was extracted from up to 250 μL of saliva via the DNeasy Power Soil Pro Kit (Qiagen, Manchester, UK) according to the manufacturer’s instructions. Following extraction, the DNA was amplified by PCR using the 515F (5’-GTGYCAGCMGCCGCGGTAA-3) and 806R (5’-GGACTACNVGGGTWTCTAAT-3) primers to amplify the variable region 4 (V4) of the 16S rRNA gene. Extracted DNA was added at a volume of 2.5 μL, followed by 5 μL of 515F primer, 5 μL of 806R primer and 12.5 μL of KAPA HiFi HotStart ReadyMix (Roche, London, UK) with a final PCR reaction volume of 25 μL. Following a denaturing step at 95°C for 3 min, PCR was performed with 25 cycles of 95°C for 30 seconds, 55°C for 30 seconds and 72°C for 30 seconds, followed by a final elongation step of 72°C for 5 min. Samples were submitted to Deep Seq Next Generation Sequencing Facility at the University of Nottingham (Nottingham, UK) for sequencing via Illumina MiSeq.

#### 2.1.2 Bioinformatics

Paired-end sequence data were imported into QIIME2 version 2022.2 (Bolyen et al., 2019) via the Casava 1.8 paired-end demultiplexed fastq format. From demultiplexed sequences, before denoising, there were 7,833,700 forward and reverse reads (median: 45,495, range: 5,502-78,889 per sample). The amplicon sequence variants generated (following chimera removal as part of the DADA2 pipeline) were aligned using multiple assignments using fast Fourier transform (mafft) (Katoh et al., 2002) and a phylogeny was constructed using FastTree2 (Price et al., 2010) for use in diversity analysis. After denoising, 6,159,441 reads remained with 1,980 ASVs identified. Rarefaction (subsampling without replacement) to an even depth of 20,925 was chosen to maximize depth without the removal of large proportions of samples, 165 (97.63%) samples and 3,452,625 (55.96% of) ASVs remained for diversity analyses (Recipients: 130 samples, Donors: 35 samples). After rarefaction, alpha and beta diversity metrics were estimated by q2-diversity (Bolyen et al., 2019). Alpha diversity was measured via Shannon’s Diversity Index (Shannon, 1948), Pielou’s Evenness (Pielou, 1975), Faith’s Phylogenetic Diversity (Faith, 1992) and the Observed ASVs (Whittaker, 1960; Whittaker, 1972). Significant differences in alpha-diversity metrics between groups were tested using Kruskal-Wallis one-way analysis of variance (ANOVA) tests (and, where significant, with Dunn’s Multiple Comparison test) within GraphPad Prism 9 (GraphPad Software, California, US) and plotted using the same software. To further assess the variation in alpha diversity caused by (donor or recipient) groups, timepoints and the interaction between them a two-way ANOVA was performed within QIIME2. For beta diversity analysis, four metrics applied to each dataset before analysis were: Jaccard (Jaccard, 1912), Bray-Curtis (Bray and Curtis, 1957), unweighted UniFrac, and weighted UniFrac (Lozupone and Knight, 2005; Lozupone et al., 2007). The clustering of beta diversity metrics between these groups was tested via PERMANOVA (999 permutations) within QIIME2 (Anderson, 2001) and the principal coordinates analysis (PCoA) plots were generated in RStudio (RStudio Team, 2020) using the qiime2R (Bisanz, 2018), phyloseq (McMurdie and Holmes, 2013) and tidyverse (Wickham et al., 2019) packages. Influence of factors and estimates of their variation in beta diversity was calculated using adonis implemented within QIIME2 from the vegan R package (Oksanen et al., 2013). Taxonomy was assigned using the q2-feature-classifier (Bokulich et al., 2018) classify-sklearn Naïve Bayes taxonomic classifier against the Greengenes 13_8 97% OTUs reference sequences (McDonald et al., 2012). Stacked taxonomic bar plots were produced using the ggplot2 package (Wickham, 2016) in RStudio (RStudio Team, 2020). Following the recommendations of Nearing et al. (2022), three differential abundance testers were selected to look for differentially abundant taxa. Differential abundance testing was performed within QIIME2 via ANCOM (Mandal et al., 2015), ALDEx2 (Gloor, 2015) using the q2-aldex2 plugin (Fernandes et al., 2013; Fernandes et al., 2014). The third differential abundance tester was the DESeq2 R package (Love et al., 2014), which was chosen due to its high sensitivity in small datasets (Weiss et al., 2017).

## 3 Results

### 3.1 Study population

The recipient cohort (n = 45) was 57.78% male with a mean BMI of 25.8 and an age of 49.8. Around one quarter (24.44%) self-reported as smokers and 22.22% as diabetic. Whilst 37.78% of transplants were pre-emptive, 62.22% of recipients were receiving some form of renal replacement therapy prior to transplant (51.11% receiving haemodialysis only, 8.89% receiving peritoneal dialysis only and one recipient (2.22%) receiving both). Immunosuppression in the recipient cohort involved two phases of therapy – induction and maintenance. For induction therapies, one recipient (2.22%) received Alemtuzumab, one recipient (2.22%) received prednisolone (due to anaphylaxis on basiliximab) and 91.11% received basiliximab (data not available for two recipients). For maintenance therapy, one recipient (2.22%) received Mycophenalate mofetil alone, one recipient (2.22%) received prednisolone with Tacrolimus and 95.56% received Mycophenalate mofetil with Tacrolimus. The donor cohort (n = 9) was 31.58% male, with an average BMI of 27.38 and age of 45.12. No donor had diabetes mellitus, one (5.26%) however self-reported smoking.

### 3.2 Relative abundance of genera

**Figure 1:**
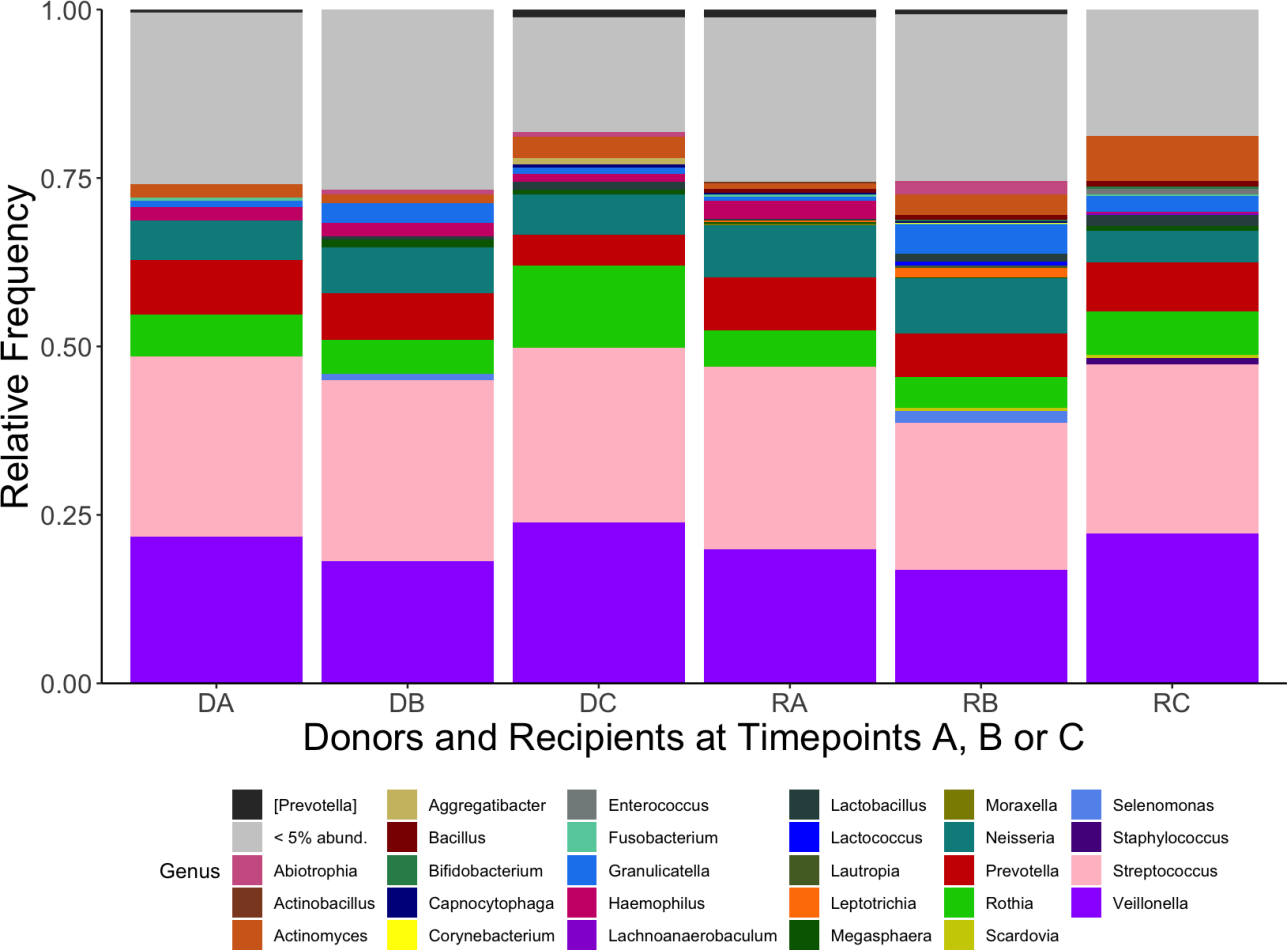
Mean relative abundance of genera from kidney transplant donors at timepoints (A–C) (DA, DB, DC) and recipients (RA, RB, RC). Each genus is represented by a different colour (legend, bottom). Genera found at relative abundance below 5% are grouped together (grey).

**Figure 2:**
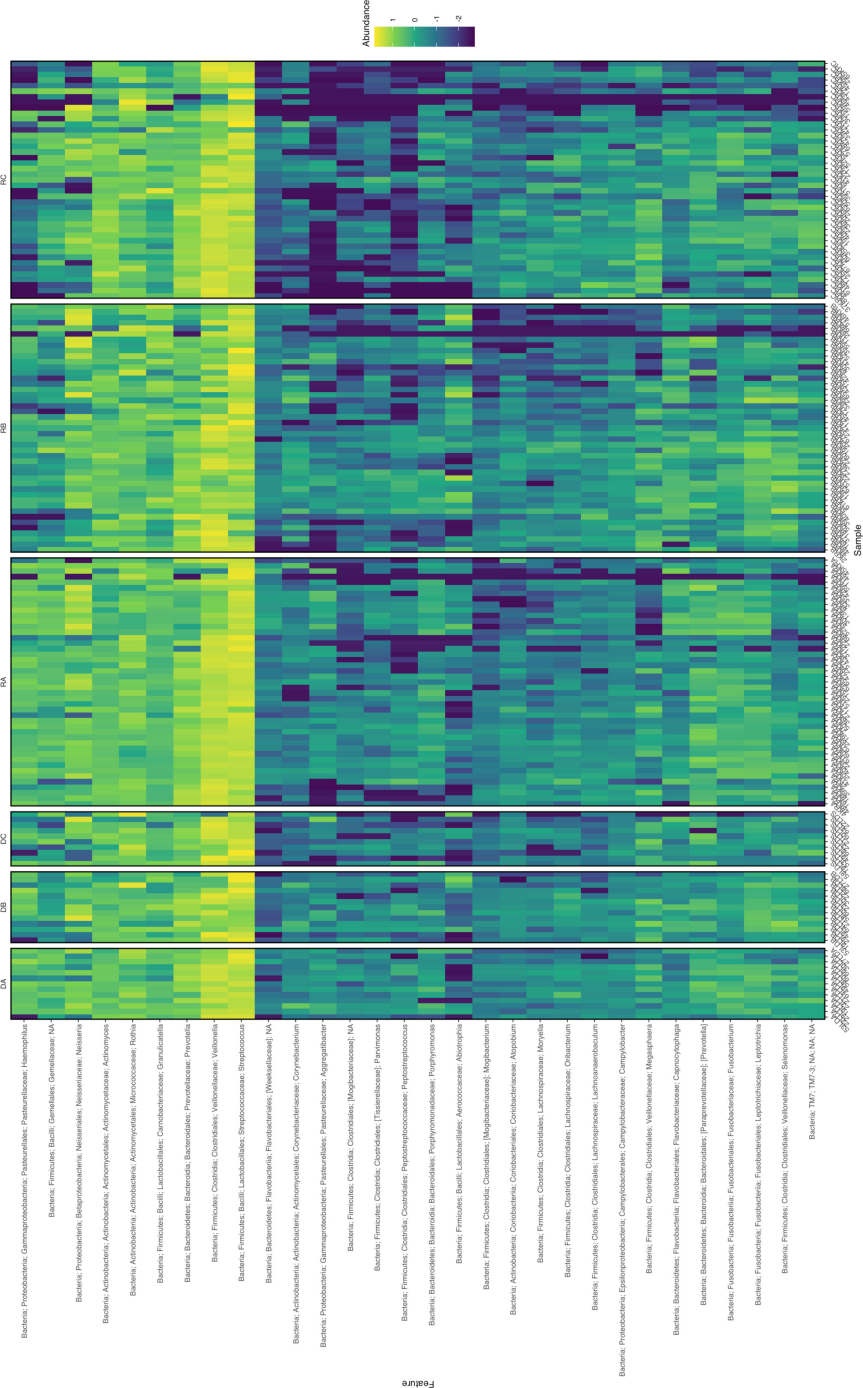
Heatmap of the 30 most abundant ASVs from oral microbiome samples of kidney transplant donors at timepoints (A–C) (DA, DB, DC) and recipients (RA, RB, RC). Abundance given as log_10_(%). Where defined, taxonomy is given on y-axis (NA at taxonomic levels unable to define). Sample identifiers are provided on the x-axis.

The 10 genera with the highest cumulative relative abundance in this study are displayed in **Table 1**. These genera (in order of highest sum abundance) included *Streptococcus*, *Veillonella*, *Rothia*, *Prevotella*, *Neisseria*, *Actinomyces*, *Granulicatella*, *Haemophilus*, *Leptotrichia* and [Prevotella] [where *Prevotella* in square brackets denotes recommended but not verified taxonomies in the Greengenes database (DeSantis et al., 2006; McDonald et al., 2012)]. The high abundance of these taxa is demonstrated in **Figure 1**, which also shows a decrease in the relative abundance of genera below 5% at timepoint C for both donors and recipients. The abundance of ASVs in donors and recipients at each timepoint is further displayed in **Figure 2**, where low abundance of ASVs belonging to *Haemophilus*, *Aggregatibacter* and *Peptostreptococcus* in recipients at timepoint C is particularly evident.

**Table 1:** The 10 defined genera with the highest combined relative abundance.

Genera	DA (%)	DB (%)	DC (%)	RA (%)	RB (%)	RC (%)
*Streptococcus*	25.63	25.47	24.17	25.41	20.53	24.46
*Veillonella*	19.85	18.24	23.32	18.52	16.66	21.41
*Rothia*	7.04	6.73	12.25	6.19	5.80	8.04
*Prevotella*	8.45	8.18	5.65	8.03	7.49	7.84
*Neisseria*	5.23	6.48	6.61	8.14	9.04	5.28
*Actinomyces*	3.91	3.55	4.37	3.27	4.22	6.95
*Granulicatella*	2.97	3.86	2.51	2.94	4.96	3.63
*Haemophilus*	4.03	3.01	1.65	4.21	1.15	0.87
*Leptotrichia*	1.80	2.26	0.69	1.83	2.79	1.03
[Prevotella]	1.69	1.10	1.89	2.57	1.41	0.94

Relative abundance (%) of each genus in each timepoint (A, B and C) for donors (D) and recipients (R) is shown. Prevotella in square brackets denotes recommended but not verified taxonomies in the Greengenes database.

### 3.3 Alpha diversity

**Figure 3:**
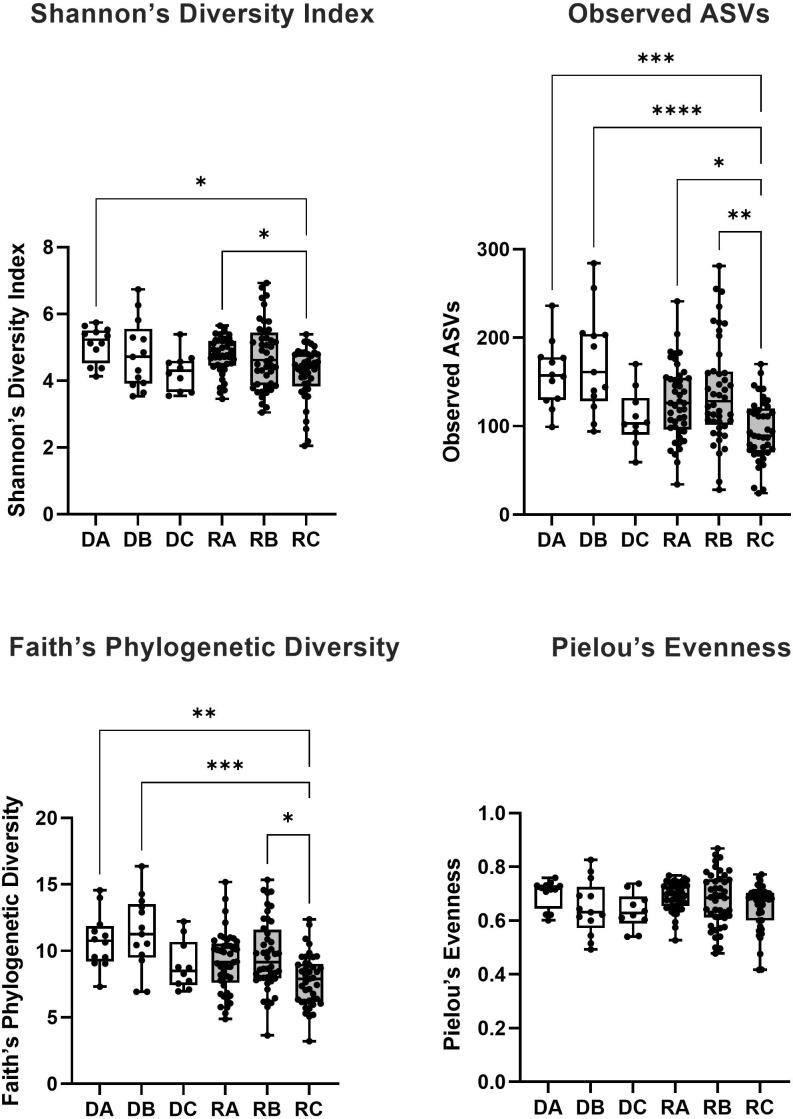
The alpha diversity of bacterial 16S rRNA sequences from saliva samples taken from kidney transplant donors (white boxes) at timepoint A (DA), B (DB) and C (DC) and recipients (grey boxes) at timepoint A (RA), B (RB) and C (RC) was measured via four metrics (Shannon’s Diversity Index, Observed ASVs, Faith’s Phylogenetic Diversity and Pielou’s Evenness). The significance of the statistical comparison (Dunn’s test) between groups is indicated above brackets where * signifies P ≤ 0.05, **P ≤ 0.01, ***P ≤ 0.001 and ****P ≤ 0.0001.

The difference in alpha diversity between donors and recipients at time points A, B and C was measured using four metrics (**Figure 3**). Comparing these groups statistically (via Kruskal-Wallis testing) revealed overall differences between mean alpha diversity using Shannon’s Diversity Index (*H*_6 =_ 18.31, p = 0.0026), the Observed ASVs metric (*H*_6 =_ 35.60, p < 0.0001) and Faith’s Phylogenetic Diversity (*H*_6 =_ 27.35, p < 0.0001). However, no significant difference was found between groups by Kruskal-Wallis testing of alpha diversity expressed via Pielou’s Evenness (*H*_6 =_ 10.05, p = 0.0739). To further investigate whether Group (Donor or Recipient), Timepoint (A, B or C) or their interaction were driving variation in alpha diversity, a two-way ANOVA was performed on each of the four metrics (**Table 2**). For all four metrics, Timepoint was found to be significant with diversity decreasing from timepoint A to C (Shannon’s Diversity Index, p <0.001, Observed ASVs, p < 0.001, Faith’s Diversity Index, p < 0.001 and Pielou’s Evenness, p = 0.021). Group (Donor or Recipient) was found to be significant, with the donor more diverse than the recipient, via the Observed ASVs metric (p = 0.002), Faith’s Phylogenetic Diversity (p = 0.001) but not via Shannon’s Diversity Index (p = 0.331), or Pielou’s Evenness (p = 0.460). The change in diversity with timepoint did not differ significantly between groups for any metric [i.e. the interaction between Group and Timepoint was not significant (**Table 2**)].

**Table 2:** Two-Way ANOVA examining the effect of Group (Donor or Recipient) and Timepoint (A, B or C) and the interaction between these terms (Group: Timepoint) on alpha diversity of oral microbiome samples.

	Sum of Squares	Degrees of freedom	F value	P value
Shannon’s Diversity Index				
Group	0.597	1	0.953	0.331
Timepoint	11.319	2	9.035	**<0.001**
Group: Timepoint	0.537	2	0.428	0.652
Residual	99.595	159		
Observed ASVs				
Group	20397.904	1	9.693	**0.002**
Timepoint	66869.587	2	15.888	**<0.001**
Group: Timepoint	1738.204	2	0.413	0.662
Residual	334595.752	159		
Faith’s Phylogenetic Diversity				
Group	62.411	1	11.543	**0.001**
Timepoint	110.134	2	10.185	**<0.001**
Group: Timepoint	1.647	2	0.152	0.859
Residual	859.678	159		
Pielou’s Evenness				
Group	0.004	1	0.549	0.460
Timepoint	0.052	2	3.974	**0.021**
Group: Timepoint	0.008	2	0.618	0.540
Residual	1.039	159		

Significance (p<0.05) is indicated in bold.

Where overall significance was found by Kruskal-Wallis, *post hoc* multiple comparisons testing was performed via Dunn’s multiple comparisons testing (**Figure 3**). For Shannon’s Diversity Index, significant differences were found between the mean alpha diversity of donors at timepoint A (5.099) and recipients at timepoint C (4.219), p adj. = 0.0102. The alpha diversity of recipients at timepoint C (4.219) was also significantly lower than recipients at timepoint A (4.759), p adj. = 0.0436. For Observed ASVs there was a significant difference in the alpha diversity of donors at timepoint A (158.5) compared with recipients at timepoint C (94.41), p adj. = 0.0003. Likewise, there was a significant difference between recipients at timepoint A (127.1) and timepoint C (94.41), p adj. = 0.0122). Recipients at timepoint B (139.0) also had significantly higher alpha diversity than at timepoint C (94.41), p adj. = 0.0012. And recipients at timepoint C also had lower alpha diversity than donors at timepoint B (172.1), p adj. <0.0001. For Faith’s Phylogenetic Diversity, donors at timepoint A (10.76) had a mean greater than recipients at timepoint C (3.208), p adj. = 0.0022. Recipients at timepoint C also had a significantly lower mean (3.208) than recipients at timepoint B (9.638), p adj. = 0.0155 and donors at timepoint B (11.34), p adj. = 0.0004. All other means were not significantly different to one another (p adj. > 0.05).

### 3.4 Beta diversity

**Figure 4:**
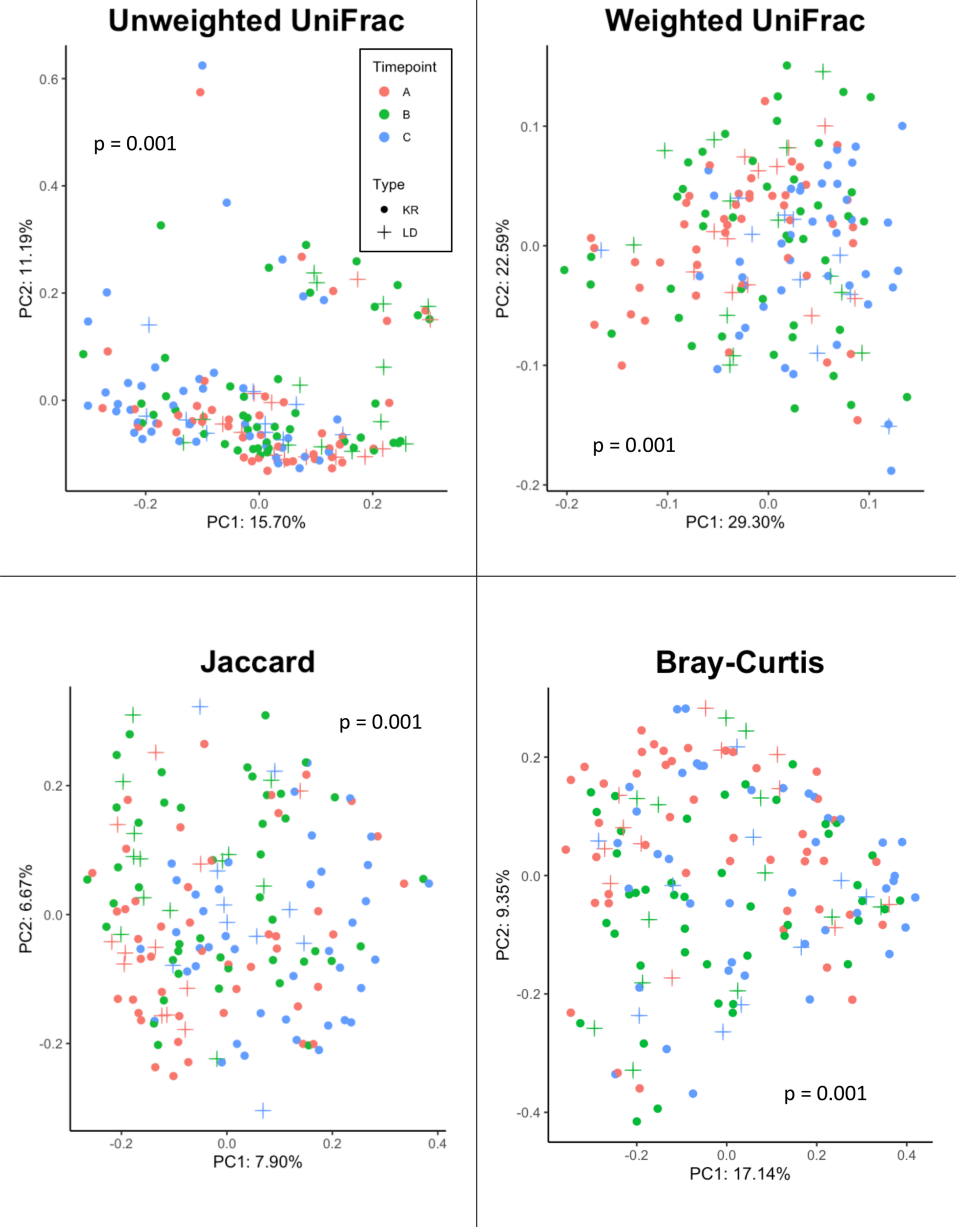
Beta diversity analysis of samples from Donor and Recipients (Recipients = circle symbols, Donors = cross symbols) at Timepoints (A) (red symbols), (B) (green symbols) and (C) (blue symbols) measured via four metrics (Jaccard, Bray-Curtis, Unweighted UniFrac, and Weighted UniFrac). Significance (p) relative to overall PERMANOVA testing is indicated for each metric.

Beta-diversity was measured using four distance metrics: Jaccard, Bray-Curtis, Unweighted UniFrac and Weighted UniFrac. Differential clustering according to beta diversity for each of these metrics was confirmed by overall PERMANOVA, which showed that donors and recipients at timepoints A, B and C were significantly distinct by Unweighted UniFrac (*F*=2.4, p=0.001), Weighted UniFrac (*F*=3.0, p=0.001), Jaccard (*F*=1.9, p=0.001) and Bray-Curtis (*F*=2.5, p=0.001) (see **Figure 4**). Following confirmation of overall significance, pairwise PERMANOVA (999 permutations) (**Supplementary Table 1**) on each of the four metrics revealed significant differences between donor and recipient timepoints. Comparisons which were found to be significantly different by all four metrics included: (i) donors at timepoint A and recipients at timepoint C, (ii) donors at timepoint B and recipients at timepoint C, (iii) recipients at timepoint A and timepoint B, (iv) recipients at timepoint A and timepoint C and (v) recipients at timepoint B and timepoint C.

Adonis was also applied to assess the statistical significance between each of the four beta diversity metrics and sample classifications based on Group (Donor or Recipient) and Timepoint (A, B or C), as well as their interaction (**Table 3**). For Unweighted UniFrac, both Group (p = 0.005) and Timepoint (p = 0.001) were significant, accounting for 1.5% and 4.7% (R^2 =^ 0.015, 0.047) of the observed variation, respectively. Likewise, using Jaccard and Bray-Curtis, both Group (Jaccard: p = 0.001, R^2 ^= 0.014, Bray-Curtis: p = 0.044, R^2 ^= 0.010) and Timepoint (Jaccard: p = 0.001, R^2 ^= 0.034, Bray-Curtis: p = 0.001, R^2 ^= 0.055) were found to be significant drivers of variation. For weighted UniFrac, only Timepoint was found to be significant (p = 0.001, R^2 ^= 0.071), with Group not found to be significant (p = 0.554). No metric found the interaction between Group and Timepoint to be significant (Unweighted UniFrac, p = 0.783, Weighted UniFrac, p = 0.567, Jaccard, p = 0.997, Bray-Curtis, p = 0.938).

**Table 3:** PERMANOVA (999 permutations) comparing the influence of Group (Donor or Recipient) and Timepoint (A, B or C) as well as the interaction of these terms (Group: Timepoint) on beta diversity of oral microbiome samples.

	Df	SumsOfSqs	MeanSqs	F.Model	R^2^	P value
Unweighted UniFrac						
Group	1	0.337	0.337	2.593	0.015	**0.005**
Timepoint	2	1.039	0.519	3.999	0.047	**0.001**
Group: Timepoint	2	0.212	0.106	0.815	0.010	0.783
Residuals	159	20.655	0.130	–	0.929	–
Total	164	22.242	–	–	1.000	–
Weighted UniFrac						
Group	1	0.012	0.012	0.765	0.004	0.554
Timepoint	2	0.188	0.094	6.176	0.071	**0.001**
Group: Timepoint	2	0.026	0.013	0.843	0.010	0.567
Residuals	159	2.423	0.015	–	0.915	–
Total	164	2.649	–	–	1.000	–
Jaccard						
Group	1	0.659	0.659	2.349	0.014	**0.001**
Timepoint	2	1.591	0.796	2.838	0.034	**0.001**
Group: Timepoint	2	0.416	0.208	0.743	0.009	0.997
Residuals	159	44.568	0.280	–	0.944	–
Total	164	47.234	–	–	1.000	–
Bray-Curtis						
Group	1	0.380	0.380	1.689	0.010	**0.044**
Timepoint	2	2.129	1.064	4.736	0.055	**0.001**
Group: Timepoint	2	0.304	0.152	0.676	0.008	0.938
Residuals	159	35.735	0.225	–	0.927	–
Total	164	38.547	–	–	1.000	–

Significance (p value <0.05) is indicated in bold.

### 3.5 Changes in ASVs over time

#### 3.5.1 Change in recipients within 1 day post-transplant: (Recipients at timepoints A vs B)

Differential abundance testing was employed to compare the expression of ASVs in recipients at different timepoints. Comparing timepoints A and B for recipients via DESeq2 revealed 10 significantly different ASVs, belonging to the genera *Abiotrophia*, *Actinobacillus* (including *A. parahaemolyticus*), *Actinomyces*, *Fusobacterium*, *Granulicatella*, *Haemophilus* (including *H. parainfluenzae*), and *Veillonella*, and an unclassified genus belonging to the family Gemellaceae (**Supplementary Table 2**). The same comparison, performed using ALDEx2, found 6 ASVs to be in differential abundance, belonging to the genera (where classified): *Streptococcus*, *Haemophilus* (including *H. parainfluenzae*), *Actinobacillus*, *Granulicatella* and an unclassified genus belonging to Gemellaceae (**Supplementary Table 3**). ANCOM detected four ASVs in differential abundance between timepoints A and B, which belonged to the genera: *Haemophilus* (ASV ID: ad4491df2aa2a9d107efeb75973869b9, W = 1453, clr = 2.799), *Granulicatella* (ASV ID: 1608689532ec031cfd9807c7dfe325a2, W = 1404, clr = 2.091), family Gemellaceae (genus unclassified, ASV ID: a2397abbce9d4a1bbf9d45e8c61edbf3, W = 1375, clr = 1.848) and *Actinobacillus* (ASV ID: 9ca30664548a41cccc4964bf6be8cc9c, W = 1350, clr = 2.082) (positive clr value indicates greater relative abundance at timepoint A). These four ASVs were detected as differentially abundant by all three methods and are, therefore, consensus results.

#### 3.5.2 Change in recipients within 1 week post-transplant (Recipients at timepoints A vs C)

Comparing samples taken from recipients at timepoint A with those taken later at timepoint C, DESeq2 revealed 15 significantly different ASVs (**Supplementary Table 2**), belonging to *Actinobacillus*, *Actinomyces, Aggregatibacter* (*A. segnis*), *Capnocytophaga*, *Haemophilus*, [Prevotella], *Peptostreptococcus*, *Peptococcus*, *Streptococcus* and unclassified genera belonging to the Orders CW040 and Clostridiales, and Families [Weeksellaceae] and Lachnospiraceae. Using ALDEx2, 9 ASVs were found to be in differential abundance, belonging to *Actinobacillus*, *Aggregatibacter* (*A. segnis*), *Granulicatella*, *Haemophilus* (including *H. parainfluenzae*), *Streptococcus*, *Peptococcus* and an unclassified genus belonging to the family [Weeksellaceae] (**Supplementary Table 2**). ANCOM detected six ASVs in differential abundance, which belonged to *Haemophilus* (ASV ID: ad4491df2aa2a9d107efeb75973869b9, W = 1283, clr = 3.073) and *H. parainfluenzae* (ASV ID: 5ab210b70bbb9d11943b629b9c1adea3, W = 1283, clr = 3.273), *Aggregatibacter segnis* (ASV ID: eff784a8bb5224c68442ad155be4fc32, W = 1259, clr = 2.523), *Peptostreptococcus* (ASV ID: fa3c31c44460408b8a4f40ec20ac7911, W = 1248, clr = 2.411), *Actinobacillus* (ASV ID: 9ca30664548a41cccc4964bf6be8cc9c, W = 1212, clr = 2.242) and an unclassified genus belonging to the family [Weeksellacaea] (ASV ID: 609b93fa4edf56f39d7a8c2d888b99b1, W = 1164, 1.779) (positive clr value indicates increased relative abundance at timepoint A). Each of the differentially expressed ASVs detected by ANCOM were also found by ALDEx2 and DESeq2, hence, were consensus results.

#### 3.5.3 Change in donors within 1 day post-transplant: (Donors at timepoints A vs B)

DESeq2 comparison of donors at timepoints A and B revealed only one ASV in differential abundance, belonging to *Actinobacillus parahaemolyticus* (**Supplementary Table 2**). No ASVs were found to be differentially abundant using ALDEx2 or ANCOM between these timepoints.

#### 3.5.4 Change in donors within 1 week post-transplant: (Donors at timepoints A vs C)

DESeq2 comparison of donors at timepoints A and C found four ASVs in differential abundance, including *Aggregatibacter*, *Fusobacterium*, *Veillonella* and an unclassified genus belonging to the order CW040 (**Supplementary Table 2**). ANCOM found one ASV in (increased) relative abundance belonging to *Bulleidia moorei* (ASV ID: 38c395eb625ea0e22580f2719dd368f0, W = 180, clr = -2.093) at timepoint A (**Supplementary Table 3**). However, ALDEx2 found no differentially abundant ASVs between the time points. There was, therefore, no consensus ASVs detected by all testers.

## 4 Discussion

We have profiled the microbiota of kidney donors and recipients longitudinally in the seven days surrounding kidney transplantation, with comparisons between timepoints and donor/recipient groups investigated in terms of alpha and beta diversity, and composition of ASVs.

### 4.1 Donors and recipients show similar diversity patterns during first week post-transplant

No significant difference in oral alpha diversity was reported in a cross-sectional comparison of donors and recipients (Diaz et al., 2013). Whereas in a longitudinal study of recipients only, alpha diversity (Shannon’s Index) was reportedly decreased in oral sample over time (Fricke et al., 2014). The present study combined cross sectional and longitudinal sampling techniques to add several key observations to the findings of these studies. In recipients, general significance patterns implied a trend of decreasing alpha diversity in the first week after kidney transplant. For the smaller donor cohort, this pattern was also visible, but not numerically significant in these analyses. Using this approach, no significant change in the alpha diversity of donors between any time points was detected within the group via Kruskal Wallis posthoc testing. Patterns of alpha diversity of donors and recipients were similar (**Figure 3**). Performing a two-way ANOVA showed that (in all metrics) timepoint and (using Observed ASVs and Faith’s Phylogenetic Diversity only) group (donor or recipient) were significant factors driving variation in alpha diversity, however, the interaction of group and timepoint was not found to be significant using any metric. Lack of significance in the interaction term suggests that donors and recipients do follow somewhat similar patterns in this period, with the lower sample size in donors preventing this from being detected significantly in ensuing post-hoc analysis following Kruskal-Wallis. The cause of donor alpha diversity falling in this period would be unclear since prophylactic antibiotics are not necessarily prescribed for donors and could point towards an effect of surgery alone as a driver of rapid reduced alpha diversity in the oral microbiome. Donors at timepoint A did have significantly higher alpha diversity compared to recipients at timepoint C by three out of four metrics (Shannon’s, Observed and Faith’s), highlighting the deviation of the post-transplant microbiome from the pre-transplant state. The significant interaction reported between donors at timepoint B and recipients at timepoint B (via Observed and Faith’s metrics), also highlights this trend.

There were no comparisons found to be significantly different via Pielou’s Evenness. This metric takes into account species evenness, which refers to how close in number (e.g. relative abundance) ASVs are in the environmental samples (Pielou, 1975). Since this metric was not different between measurements it is possible to infer the decrease in alpha diversity seen in recipients post-transplant is influenced by a reduction in the number of ASVs observed (community richness) rather than the proportion of those ASVs (community evenness).

Overall, PERMANOVA testing of these four metrics revealed a significant difference in the beta diversity according to donor or recipient time points via all four metrics. Following PERMANOVA, post-hoc pairwise testing showed that recipients at time points A, B and C were significantly different in every metric and every combination of pairwise comparisons. Given the combination of features measured by each of the four metrics, this result shows a shifting beta diversity profile both one day and one week after transplant for recipients, including changes in relative abundance, unique phylogeny and compositional dissimilarity. Differences in beta diversity were found between donor samples at time points A and C (via Jaccard only) and B compared with C (via Unweighted only). This suggests that kidney donation in the absence of immunosuppressive regimens could cause detectable, longitudinal shifts in the oral microbiota in terms of beta diversity.

Donors at timepoint B were also significantly different from recipients at timepoint A (Unweighted, Jaccard, Bray-Curtis) and C (all metrics). Donors at timepoint C were significantly different to recipients at the same time point using only the Jaccard metric. Notably, there was no difference between donors and recipients in terms of any beta diversity metric at time points A or B, and in only one out of four measures (Jaccard) at time point C. In summary, this could imply that donors and recipients show similar patterns of longitudinal beta-diversity change post-transplant, suggesting the physiological pressures on the oral microbiome of surgery are broadly similar and the impact of returning to ‘normal’ renal function on the oral microbiota is comparable. Indeed, according to the adonis testing the interaction of Group and Timepoint, no significant difference was found using any of the four metrics, hence significant variation driven by the interaction of donors and recipients was not found.

### 4.2 Decrease in ASVs associated with infective endocarditis in recipients

Differential abundance testing was performed between donors and recipients at different time points to detect ASVs which were differentially expressed within the first week post-transplant. Per the recommendations of Nearing et al. (2022), three differential abundance testers were used on this dataset to seek consensus results. In doing so, comparisons of recipients prior to transplant, and the day afterwards, revealed four consensus ASVs in differential abundance. Three of these ASVs were lower in abundance following transplant and belonged to *Haemophilus*, *Granulicatella* and *Actinobacillus*. One ASV belonging to the family Gemellaceae increased one day after the transplant. The family Gemellaceae is a normal constituent of the oral microbiome and has previously been associated with a healthy (compared with halitosis) state in the oral cavity (Seerangaiyan et al., 2017). Interpreting a result, such as an apparent increase in commensal bacteria, can be confounded by limitations of relative abundance data. For example, an apparent increase in the relative abundance of an ASV could be caused by an increase in its total abundance, or a decrease in the total abundance of other ASVs (Rao et al., 2021). Thus, whether an ASV increasing in abundance is “blooming, or others are dying out” is an effect difficult to untangle in the absence of absolute abundance data (Rao et al., 2021). Three species of *Granulicatella* have been described *(G. adiacens*, *G. elegans* and *G. balaenopterae)* (Collins and Lawson, 2000). The genus was originally grouped as ‘nutritionally variant streptococci’ (Frenkel and Hirsch, 1961) and despite being a normal component of the oral flora, has been associated with invasive infection and bacterial endocarditis (Cargill et al., 2012). Similarly, *Actinobacillus* also has a role in infective endocarditis (Paturel et al., 2004). Their apparent decrease in relative abundance following transplant would contrast with previous observations of increased opportunistic pathogens post-transplant (Diaz et al., 2013; Fricke et al., 2014). Taken together, a reduction in these genera could represent a lower risk of bacterial endocarditis, a rare but high mortality complication (Moshkani Farahani et al., 2014; Tamzali et al., 2021), in the post-transplant recipient group (Ioannou et al., 2021).

Between recipients pre-transplant and the timepoint at one-week post-transplant, differential abundance testing identified 6 consensus ASVs. These ASVs included two of those identified between timepoints A and B: those belonging to *Haemophilus* and *Actinobacillus* were found to be lower in both postoperative time points (B and C) compared with preoperative timepoint A.

Lower relative abundance of a *Haemophilus* operational taxonomic unit has previously been reported following kidney transplantation (Fricke et al., 2014). In the present study, two ASVs belonging to *Haemophilus* (*H. parainfluenzae* and an unclassified species) were found to be in significantly lower abundance one week post-transplant. Like *Actinobacillus*, *Haemophilus* is also associated with endocarditis, as well as bacteraemia (Berge et al., 2021). *Aggregatibacter segnis* was also found in decreased abundance and is closely related to *Haemophilus*, originally described as *Haemophilus* before transfer to the genus *Aggregatibacter* in 2006 (Kilian, 1976; Norskov-Lauritsen and Kilian, 2006; Nørskov-Lauritsen, 2014). The similar phenotype of *A. segnis* to *H. parainfluezae* (Dewhirst et al., 1992; Olsen et al., 2005; Norskov-Lauritsen and Kilian, 2006; Nørskov-Lauritsen, 2014) could be responsible for their concomitant decrease, with similar selective pressures causing the same effect on their abundance. Like *Actinobacillus*, *Haemophilus* and *Granulicatella*, *A. segnis* is also a (rare) cause of infective endocarditis (Bangsborg et al., 1988; Somers et al., 2003; Chambers et al., 2013; Nørskov-Lauritsen, 2014). Other ASVs decreasing in relative abundance during this period were *Peptostreptococcus* and an unclassified genus belonging to the family Weeksellaceae. Like the other genera discussed here, *Peptostreptococcus* is a normal member of the oral flora with causative links with infective endocarditis (Capunitan and Conte, 2010).

For patients on haemodialysis, the risk of infective endocarditis has been recognised since the 1960s (Doulton et al., 2003) and patients with infective endocarditis were estimated to be 16.9 times more likely to have suffered severe kidney disease (Strom et al., 2000; Naqvi and Collins, 2006). Hence, elevated levels of infective endocarditis-associated genera could have an involvement in this risk to pre-transplant cohorts. In CKD, differences in oral microbiota are associated with dialysis (Araújo et al., 2015; Simões-Silva et al., 2018) as well as uraemia and the build-up of urea causing increased oral pH (Casiano-Colón and Marquis, 1988; Wijeyeweera and Kleinberg, 1989; Chen et al., 1996; Morou-Bermudez and Burne, 1999; Spolidorio et al., 2006; Yaling et al., 2006; Nascimento et al., 2009; Campbell et al., 2020). Conditions in the chronic kidney disease (pre-transplant) state might create a more favourable environment for genera associated with infective endocarditis, or the effect of transplant (such as the antibiotic properties of macrolide antibiotic immunosuppressive agents) might cause their reduction in abundance. This represents an interesting hypothesis for further study.

Comparing donors at pre-donation timepoint A and one day-post donation timepoint B found no consensus ASVs in differential abundance, with only one ASV found by the highly sensitive DESeq2 test (Love et al., 2014; Weiss et al., 2017). Likewise, no consensus ASVs were found in differential abundance between donors at timepoint A or timepoint C. Together, this reveals the longitudinal stability of the oral microbiome of living kidney donors before and within one week after undergoing surgery. Within healthy individuals, the oral microbiota remains stable in health (Lazarevic et al., 2010). This result suggests that perturbations associated with hospital stay and surgery alone did not alter the relative abundance of oral genera in these normal individuals. Furthermore, it could isolate the effects seen in the recipient group as “recipient only” effects, suggesting that the role of immunosuppressive agents, removal of chronic kidney disease (and uraemia) or cessation of dialysis might be more likely to drive the observations in the recipient cohort.

Direct comparison of temporal changes in the oral microbiota of donors does have notable limitations. For example, the number of recipients from which a pre-transplant sample was taken (43) was considerably larger than the size of the donor group (13). The variance of each alpha diversity metric and differing sample sizes among groups could, for example, influence results such as ANOVA. Additionally, the differential abundance tester DESeq2, which is established to show high sensitivity with small sample groups (e.g. those with ≤20 samples per group) (Love et al., 2014; Weiss et al., 2017) found only one ASV in differential abundance between timepoints A and B, and four between A and C for donors. However, the high degree of variability between denoisers meant this study only focuses on consensus results from three analysis methods (Nearing et al., 2022).

Another significant limitation of the current study was the separation in samples taken at timepoint C for donors and recipients. To take this sample as late as possible, recipients were sampled at their first clinic visit (usually day 7) post-transplant. However, since donors do not attend similar clinic visits, the latest timepoint which could be sampled was before discharge (days 3-5). Hence, timepoint C for donors was shorter term than for recipients. Change in recipients, however, is apparent from day 1 post-transplant with four consensus ASVs detected. By contrast, stability is shown in the early stages for donors (no consensus ASVs detected) and it could be considered unlikely to expect significant changes between days 5-7.

## 5 Conclusions

The oral microbiome is detectably altered by kidney transplantation, and this has previously presented as longitudinal reductions in diversity and the increased growth of opportunistic pathogens (Diaz et al., 2013; Fricke et al., 2014). Here, to investigate how early this effect is observed, we investigated the oral microbiome of kidney transplant recipients within the first week after transplantation. Doing so revealed that alpha diversity decreased within the first week post-transplant in recipients. We did not observe increases in consensus ASVs associated with opportunistic pathogenicity or extra-oral infection during this period. Several ASVs which significantly decreased in relative abundance have been associated with infective endocarditis. Infective endocarditis is an associated risk for (pre-transplant) patients undergoing dialysis and/or with kidney disease. There are limited reports of bacterial endocarditis after kidney transplantation (Moshkani Farahani et al., 2014; Tamzali et al., 2021). Whether the observed decrease in the relative abundance of these ASVs is associated with dialysis and disease removal or transplant-associated should be an area of future investigation.

By investigating a donor cohort as well as recipients, we identified similar patterns in alpha and beta diversity, with no significant differences in their change over time between the groups. This highlights the importance of such controls and the physiological pressures on the oral microbiome associated with surgery and a hospital stay in themselves. Nonetheless, we only found consensus changes of particular ASVs in the recipient group. The reduced relative abundance of these ASVs makes them candidate targets for the antibiotic action of macrolide immunosuppressive agents and bacteria within the oral microbiome sensitive to the removal of dialysis and kidney disease state in kidney transplantation.

## Data availability statement

The data presented in the study are deposited in the NCBI SRA repository, accession number PRJNA1015422.

## Ethics statement

The studies involving humans were approved by Health Research Authority (HRA) and study protocol and documentation were given favourable ethical approval from the local research ethics committee (REC reference: 19/NW/0100). The studies were conducted in accordance with the local legislation and institutional requirements. The participants provided their written informed consent to participate in this study.

## Author contributions

PC: Conceptualization, Formal analysis, Investigation, methodology, Writing – original draft, Writing – review & editing, Data curation. TW: Formal analysis, Investigation, Methodology, Writing – original draft, Writing – review & editing. GH: Formal analysis, Methodology, Writing – review & editing, Project administration, Visualization. OP: Writing – review & editing, Investigation. hc: Investigation, Writing – review & editing. AS: Investigation, Writing – review & editing, Project administration, supervision. JK: Writing – review & editing, Funding acquisition. CK: Funding acquisition, Writing – review & editing, Conceptualization, Formal analysis, Investigation, Methodology, Supervision. TA: Conceptualization, Funding acquisition, Methodology, Writing – review & editing. AM: Conceptualization, Funding acquisition, Methodology, Writing – review & editing, Formal analysis, Investigation, Project administration, Supervision, Writing – original draft.

## References

[B1] Anderson M. J. (2001). A new method for non-parametric multivariate analysis of variance. Austral Ecol. 26, 32–46. doi: 10.1111/J.1442-9993.2001.01070.PP.X

[B2] Araújo M. V. F. Hong B.-Y. Fava P. L. Khan S. Burleson J. A. Fares G.. (2015). End stage renal disease as a modifier of the periodontal microbiome. BMC Nephrol. 16, 1–7. doi: 10.1186/s12882-015-0081-x PMC4460699 26055269

[B3] Bangsborg J. M. Tvede M. Skinhøj P. (1988). Haemophilus segnis endocarditis. J. Infect. 16, 81–85. doi: 10.1016/S0163-4453(88)96227-5 3367060

[B4] Berge A. Morenius C. Petropoulos A. Nilson B. Rasmussen M. (2021). Epidemiology, bacteriology, and clinical characteristics of HACEK bacteremia and endocarditis: a population-based retrospective study. Eur. J. Clin. Microbiol. Infect. Dis. 40, 525–534. doi: 10.1007/s10096-020-04035-y 32944895 PMC7892745

[B5] Bisanz J. E. (2018). qiime2R: Importing QIIME2 artifacts and associated data into R sessions. Accessed Dec. 2022. Available at: https://github.com/jbisanz/qiime2R, Version 0.99, 13.

[B6] Bliven K. Snow K. Carlson A. Yeager S. Kenyon N. Smith L.. (2018). Evaluating a change in surgical antibiotic prophylaxis in kidney transplant recipients. Cureus 10 (10), e3433. doi: 10.7759/cureus.3433 30546980 PMC6289557

[B7] Bokulich N. A. Kaehler B. D. Rideout J. R. Dillon M. Bolyen E. Knight R.. (2018). Optimizing taxonomic classification of marker-gene amplicon sequences with QIIME 2’s q2-feature-classifier plugin. Microbiome 6, 1–17. doi: 10.1186/s40168-018-0470-z 29773078 PMC5956843

[B8] Bolyen E. Rideout J. R. Dillon M. R. Bokulich N. A. Abnet C. C. Al-Ghalith G. A.. (2019). Reproducible, interactive, scalable and extensible microbiome data science using QIIME 2. Nat. Biotechnol. 37, 852–857. doi: 10.1038/s41587-019-0209-9 31341288 PMC7015180

[B9] Bray J. R. Curtis J. T. (1957). An ordination of the upland forest communities of southern Wisconsin. Ecol. Monogr. 27, 326–349. doi: 10.2307/1942268

[B10] Campbell P. M. Humphreys G. J. Summers A. M. Konkel J. E. Knight C. G. Augustine T.. (2020). Does the microbiome affect the outcome of renal transplantation? Front. Cell. Infect. Microbiol. 10. doi: 10.3389/fcimb.2020.558644 PMC7785772 33425774

[B11] Capunitan J. A. Conte H. A. (2010). Peptostreptococcus species: an unusual cause of infective endocarditis. Connecticut Med. 74 (2), 93–6. 20218045

[B12] Cargill J. S. Scott K. S. Gascoyne-Binzi D. Sandoe J. A. (2012). Granulicatella infection: diagnosis and management. J. Med. Microbiol. 61, 755–761. doi: 10.1099/jmm.0.039693-0 22442291

[B13] Casiano-Colón A. Marquis R. E. (1988). Role of the arginine deiminase system in protecting oral bacteria and an enzymatic basis for acid tolerance. Appl. Environ. Microbiol. 54, 1318–1324. doi: 10.1128/aem.54.6.1318-1324.1988 2843090 PMC202656

[B14] Chambers S. T. Murdoch D. Morris A. Holland D. Pappas P. Almela M.. (2013). HACEK infective endocarditis: characteristics and outcomes from a large, multi-national cohort. PloS One 8, e63181. doi: 10.1371/journal.pone.0063181 23690995 PMC3656887

[B15] Chen Y. Y. Clancy K. A. Burne R. A. (1996). Streptococcus salivarius urease: genetic and biochemical characterization and expression in a dental plaque streptococcus. Infect. Immun. 64, 585–592. doi: 10.1128/IAI.64.2.585-592.1996 8550211 PMC173805

[B16] Chu F. Tsang P. Chan A. Leung W. Samaranayake L. Chan T. (2000). Oral health status, oral microflora, and non-surgical periodontal treatment of renal transplant patients receiving cyclosporin A and FK506. Ann. R. Australas. Coll. Dental surgeons 15, 286–291. 11709958

[B17] Cippà P. E. Schiesser M. Ekberg H. Van Gelder T. Mueller N. J. Cao C. A.. (2015). Risk stratification for rejection and infection after kidney transplantation. Clin. J. Am. Soc. Nephrol. 10, 2213–2220. doi: 10.2215/CJN.01790215 26430088 PMC4670759

[B18] Collins M. D. Lawson P. A. (2000). The genus Abiotrophia (Kawamura et al.) is not monophyletic: proposal of Granulicatella gen. nov., Granulicatella adiacens comb. nov., Granulicatella elegans comb. nov. and Granulicatella balaenopterae comb. nov. Int. J. Sys. Evol. Microbiol. 50, 365–369. doi: 10.1099/00207713-50-1-365 10826824

[B19] Cowan J. Bennett A. Fergusson N. Mclean C. Mallick R. Cameron D. W.. (2018). Incidence rate of post-kidney transplant infection: a retrospective cohort study examining infection rates at a large Canadian multicenter tertiary-care facility. Can. J. Kidney Health Dis. 5, 2054358118799692. doi: 10.1177/2054358118799692 30224973 PMC6136109

[B20] Da Silva L. C. F. Freitas R. D. De Andrade M. P. Piva M. R. Martins P. R. S. Santos T. D. (2012). Oral lesions in renal transplant. J. Craniofacial Surg. 23, E214–E218. doi: 10.1097/SCS.0b013e31824de388 22627437

[B21] DeSantis T. Z. Hugenholtz P. Larsen N. Rojas M. Brodie E. L. Keller K.. (2006). Greengenes, a chimera-checked 16S rRNA gene database and workbench compatible with ARB. Appl. Environ. Microbiol. 72, 5069–5072. doi: 10.1128/AEM.03006-05 16820507 PMC1489311

[B22] Dewhirst F. E. Paster B. J. Olsen I. Fraser G. J. (1992). Phylogeny of 54 representative strains of species in the family Pasteurellaceae as determined by comparison of 16S rRNA sequences. J. bacteriol. 174, 2002–2013. doi: 10.1128/jb.174.6.2002-2013.1992 1548238 PMC205807

[B23] Diaz P. I. Hong B.-Y. Frias-Lopez J. Dupuy A. K. Angeloni M. Abusleme L.. (2013). Transplantation-associated long-term immunosuppression promotes oral colonization by potentially opportunistic pathogens without impacting other members of the salivary bacteriome. Clin. Vaccine Immunol. 20 (6), 920–30. doi: 10.1128/CVI.00734-12 23616410 PMC3675961

[B24] Doulton T. Sabharwal N. Cairns H. S. Schelenz S. Eykyn S. O’donnell P.. (2003). Infective endocarditis in dialysis patients: new challenges and old. Kidney Int. 64, 720–727. doi: 10.1046/j.1523-1755.2003.00136.x 12846771

[B25] Faith D. P. (1992). Conservation evaluation and phylogenetic diversity. Biol. Conserv. 61, 1–10. doi: 10.1016/0006-3207(92)91201-3

[B26] Fernandes A. D. Macklaim J. M. Linn T. G. Reid G. Gloor G. B. (2013). ANOVA-like differential expression (ALDEx) analysis for mixed population RNA-Seq. PloS One 8, e67019. doi: 10.1371/journal.pone.0067019 23843979 PMC3699591

[B27] Fernandes A. D. Reid J. N. Macklaim J. M. Mcmurrough T. A. Edgell D. R. Gloor G. B. (2014). Unifying the analysis of high-throughput sequencing datasets: characterizing RNA-seq, 16S rRNA gene sequencing and selective growth experiments by compositional data analysis. Microbiome 2, 1–13. doi: 10.1186/2049-2618-2-15 24910773 PMC4030730

[B28] Frenkel A. Hirsch W. (1961). Spontaneous development of L forms of streptococci requiring secretions of other bacteria or sulphydryl compounds for normal growth. Nature 191, 728–730. doi: 10.1038/191728a0 13701753

[B29] Fricke W. Maddox C. Song Y. Bromberg J. (2014). Human microbiota characterization in the course of renal transplantation. Am. J. Transplant. 14, 416–427. doi: 10.1111/ajt.12588 24373208

[B30] Gloor G. (2015). ALDEx2: ANOVA-Like Differential Expression tool for compositional data. ALDEX manual modular 20, 1–11.

[B31] Han Y. W. Wang X. (2013). Mobile microbiome: oral bacteria in extra-oral infections and inflammation. J. Dental Res. 92, 485–491. doi: 10.1177/0022034513487559 PMC3654760 23625375

[B32] Ioannou P. Alexakis K. Kofteridis D. P. (2021). Endocarditis in kidney transplant recipients: a systematic review. J. Chemother. 33, 269–275.10.1080/1120009x.2020.1861512. doi: 10.1080/1120009X.2020.1861512 33327869

[B33] Jaccard P. (1912). The distribution of the flora in the alpine zone. 1. New Phytol. 11, 37–50. doi: 10.1111/j.1469-8137.1912.tb05611.x

[B34] Jakobsson H. E. Jernberg C. Andersson A. F. Sjölund-Karlsson M. Jansson J. K. Engstrand L. (2010). Short-term antibiotic treatment has differing long-term impacts on the human throat and gut microbiome. PloS One 5 (3), e9836. doi: 10.1371/journal.pone.0009836 20352091 PMC2844414

[B35] Karuthu S. Blumberg E. A. (2012). Common infections in kidney transplant recipients. Clin. J. Am. Soc. Nephrol. 7, 2058–2070. doi: 10.2215/CJN.04410512 22977217

[B36] Katoh K. Misawa K. Kuma K. I. Miyata T. (2002). MAFFT: a novel method for rapid multiple sequence alignment based on fast Fourier transform. Nucleic Acids Res. 30, 3059–3066. doi: 10.1093/nar/gkf436 12136088 PMC135756

[B37] Kilian M. (1976). A taxonomic study of the genus Haemophilus, with the proposal of a new species. Microbiology 93, 9–62. 10.1099/00221287-93-1-9 772168

[B38] Korpela K. Salonen A. Virta L. J. Kekkonen R. A. Forslund K. Bork P.. (2016). Intestinal microbiome is related to lifetime antibiotic use in Finnish pre-school children. Nat. Commun. 7, 10410. doi: 10.1038/ncomms10410 26811868 PMC4737757

[B39] Lasisi T. J. Raji Y. R. Salako B. L. (2016). Salivary creatinine and urea analysis in patients with chronic kidney disease: a case control study. BMC Nephrol. 17, 10.10.1186/s12882–016-0222-x. doi: 10.1186/s12882-016-0222-x 26775026 PMC4715295

[B40] Lazarevic V. Whiteson K. Hernandez D. François P. Schrenzel J. (2010). Study of inter-and intra-individual variations in the salivary microbiota. BMC Genomics 11, 1–11. doi: 10.1186/1471-2164-11-523 20920195 PMC2997015

[B41] Levarda-Hudolin K. Hudolin T. Basic-Jukic N. Kastelan Z. (2016). ORAL LESIONS IN KIDNEY TRANSPLANT RECIPIENTS. Acta Clinica Croatica 55, 459–463. doi: 10.20471/acc.2016.55.03.15 29045773

[B42] Love M. I. Huber W. Anders S. (2014). Moderated estimation of fold change and dispersion for RNA-seq data with DESeq2. Genome Biol. 15, 1–21. doi: 10.1186/s13059-014-0550-8 PMC4302049 25516281

[B43] Lozupone C. A. Hamady M. Kelley S. T. Knight R. (2007). Quantitative and qualitative β diversity measures lead to different insights into factors that structure microbial communities. Appl. Environ. Microbiol. 73, 1576–1585. doi: 10.1128/AEM.01996-06 17220268 PMC1828774

[B44] Lozupone C. Knight R. (2005). UniFrac: a new phylogenetic method for comparing microbial communities. Appl. Environ. Microbiol. 71, 8228–8235. doi: 10.1128/AEM.71.12.8228-8235.2005 16332807 PMC1317376

[B45] Mandal S. Van Treuren W. White R. A. Eggesbø M. Knight R. Peddada S. D. (2015). Analysis of composition of microbiomes: a novel method for studying microbial composition. Microbial. Ecol. Health Dis. 26, 27663. doi: 10.3402/mehd.v26.27663 PMC4450248 26028277

[B46] McDonald D. Price M. N. Goodrich J. Nawrocki E. P. Desantis T. Z. Probst A.. (2012). An improved Greengenes taxonomy with explicit ranks for ecological and evolutionary analyses of bacteria and archaea. ISME J. 6, 610–618. doi: 10.1038/ismej.2011.139 22134646 PMC3280142

[B47] McMurdie P. J. Holmes S. (2013). phyloseq: an R package for reproducible interactive analysis and graphics of microbiome census data. PloS One 8, e61217. doi: 10.1371/journal.pone.0061217 23630581 PMC3632530

[B48] Morou-Bermudez E. Burne R. A. (1999). Genetic and physiologic characterization of urease of Actinomyces naeslundii. Infect. Immun. 67, 504–512. doi: 10.1128/IAI.67.2.504-512.1999 9916052 PMC96348

[B49] Moshkani Farahani M. Rostami Z. Einollahi B. Khosravi A. Nemati E. Lessan Pezeshki M.. (2014). Infective endocarditis after renal transplantation. Nephrourol. Mon 6, e12326. doi: 10.5812/numonthly.12326 24719812 PMC3968955

[B50] Naqvi S. B. Collins A. J. (2006). Infectious complications in chronic kidney disease. Adv. chronic Kidney Dis. 13, 199–204. doi: 10.1053/j.ackd.2006.04.004 16815225

[B51] Nascimento M. Gordan V. Garvan C. Browngardt C. Burne R. (2009). Correlations of oral bacterial arginine and urea catabolism with caries experience. Oral. Microbiol. Immunol. 24, 89–95. doi: 10.1111/j.1399-302X.2008.00477.x 19239634 PMC2742966

[B52] Nearing J. T. Douglas G. M. Hayes M. G. Macdonald J. Desai D. K. Allward N.. (2022). Microbiome differential abundance methods produce different results across 38 datasets. Nat. Commun. 13, 1–16. doi: 10.1038/s41467-022-28034-z 35039521 PMC8763921

[B53] Nørskov-Lauritsen N. (2014). Classification, identification, and clinical significance of Haemophilus and Aggregatibacter species with host specificity for humans. Clin. Microbiol. Rev. 27, 214–240. doi: 10.1128/CMR.00103-13 24696434 PMC3993099

[B54] Norskov-Lauritsen N. Kilian M. (2006). Reclassification of actinobacillus actinomycetemcomitans, Haemophilus aphrophilus, Haemophilus paraphrophilus and Haemophilus segnis as Aggregatibacter actinomycetemcomitans gen. nov., comb. nov., Aggregatibacter aphrophilus comb. nov. and Aggregatibacter segnis comb. nov., and emended description of Aggregatibacter aphrophilus to include V factor-dependent and V factor-independent isolates. Int J Syst Evol Microbiol 56 (Pt 9), 2135–2146. doi: 10.1099/ijs.0.64207-0 16957111

[B55] Oksanen J. Blanchet F. G. Kindt R. Legendre P. Minchin P. R. O’hara R.. (2013). Package ‘vegan’. Community Ecol. package ver. 2, 1–295.

[B56] Olsen I. Dewhirst F. Paster B. Busse H. (2005). “Family Pasteurellaceae Pohl 1981, 382 VP (Effective publication: Pohl 1979, 81),” in Bergey’s manual of systematic bacteriology. Eds. Brenner D. J. Krieg N. R. Staley J. T. (New York: Springer).

[B57] Orlando G. Manzia T. M. Sorge R. Iaria G. Angelico R. Sforza D.. (2015). One-shot versus multidose perioperative antibiotic prophylaxis after kidney transplantation: a randomized, controlled clinical trial. Surgery 157, 104–110. doi: 10.1016/j.surg.2014.06.007 25304836

[B58] Paturel L. Casalta J. P. Habib G. Nezri M. Raoult D. (2004). Actinobacillus actinomycetemcomitans endocarditis. Clin. Microbiol. Infect. 10, 98–118. doi: 10.1111/j.1469-0691.2004.00794.x 14759235

[B59] Pielou E. C. (1975). Ecological diversity. New York: Wiley.

[B60] Price M. N. Dehal P. S. Arkin A. P. (2010). FastTree 2–approximately maximum-likelihood trees for large alignments. PloS One 5, e9490. doi: 10.1371/journal.pone.0009490 20224823 PMC2835736

[B61] Rao C. Coyte K. Z. Bainter W. Geha R. S. Martin C. R. Rakoff-Nahoum S. (2021). Multi-kingdom ecological drivers of microbiota assembly in preterm infants. Nature 591, 633–638. doi: 10.1038/s41586-021-03241-8 33627867 PMC7990694

[B62] Regev E. Zeltser R. Lustmann J. (1992). Lip carcinoma in renal allograft recipient with long-term immunosuppressive therapy. Oral. surg. Oral. med. Oral. Pathol. 73, 412–414. doi: 10.1016/0030-4220(92)90316-I 1574300

[B63] RStudio Team (2020). RStudio: Integrated Development for R.

[B64] Sahebjamee M. Shahabi M. S. Nikoobakht M. R. Beitollahi J. M. Mansourian A. (2010). Oral lesions in kidney transplant patients. Iranian J. Kidney Dis. 4, 232–236. 20622313

[B65] Saraiva L. Lotufo R. F. Pustiglioni A. N. Silva H. T. Jr. Imbronito A. V. (2006). Evaluation of subgingival bacterial plaque changes and effects on periodontal tissues in patients with renal transplants under immunosuppressive therapy. Oral. Surg. Oral. Med. Oral. Pathol. Oral. Radiol. Endod. 101, 457–462. doi: 10.1016/j.tripleo.2005.08.004 16545709

[B66] Seerangaiyan K. Van Winkelhoff A. J. Harmsen H. J. Rossen J. W. Winkel E. G. (2017). The tongue microbiome in healthy subjects and patients with intra-oral halitosis. J. breath Res. 11, 036010. doi: 10.1088/1752-7163/aa7c24 28875948

[B67] Shannon C. E. (1948). A mathematical theory of communication. Bell system Tech. J. 27, 379–423. doi: 10.1002/j.1538-7305.1948.tb01338.x

[B68] Simões-Silva L. Araujo R. Pestana M. Soares-Silva I. Sampaio-Maia B. (2018). The microbiome in chronic kidney disease patients undergoing hemodialysis and peritoneal dialysis. Pharmacol. Res. 130, 143–151. doi: 10.1016/j.phrs.2018.02.011 29444477

[B69] Somers C. Millar B. Xu J. Moore D. Moran A. Maloney C.. (2003). Haemophilus segnis: a rare cause of endocarditis. Clin. Microbiol. Infect. 9, 1048–1050. doi: 10.1046/j.1469-0691.2003.00703.x 14616751

[B70] Spolidorio L. C. Spolidório D. M. P. Massucato E. M. S. Neppelenbroek K. Campanha N. Sanches M. (2006). Oral health in renal transplant recipients administered cyclosporin A or tacrolimus. Oral. Dis. 12, 309–314. doi: 10.1111/j.1601-0825.2005.01200.x 16700742

[B71] Stanke-Labesque F. Gautier-Veyret E. Chhun S. Guilhaumou R. (2020). Inflammation is a major regulator of drug metabolizing enzymes and transporters: Consequences for the personalization of drug treatment. Pharmacol. Ther. 215, 107627. doi: 10.1016/j.pharmthera.2020.107627 32659304 PMC7351663

[B72] Strom B. L. Abrutyn E. Berlin J. A. Kinman J. L. Feldman R. S. Stolley P. D.. (2000). Risk factors for infective endocarditis: oral hygiene and nondental exposures. Circulation 102, 2842–2848. doi: 10.1161/01.CIR.102.23.2842 11104742

[B73] Tamzali Y. Danthu C. Aubry A. Brousse R. Faucher J. F. El Ouafi Z.. (2021). High mortality and graft loss after infective endocarditis in kidney transplant recipients: A case-controlled study from two centers. Pathogens 10. doi: 10.3390/pathogens10081023 PMC8397984 34451487

[B74] Thomas D. Seddon S. Shepherd J. (1993). Systemic immunosuppression and oral Malignancy: a report of a case and review of the literature. Br. J. Oral. Maxillofac. Surg. 31, 391–393. doi: 10.1016/0266-4356(93)90197-5 8286295

[B75] Weiss S. Xu Z. Z. Peddada S. Amir A. Bittinger K. Gonzalez A.. (2017). Normalization and microbial differential abundance strategies depend upon data characteristics. Microbiome 5, 1–18. doi: 10.1186/s40168-017-0237-y 28253908 PMC5335496

[B76] Whittaker R. H. (1960). Vegetation of the Siskiyou mountains, Oregon and California. Ecol. Monogr. 30, 279–338. doi: 10.2307/1943563

[B77] Whittaker R. H. (1972). Evolution and measurement of species diversity. Taxon 21, 213–251. doi: 10.2307/1218190

[B78] Wickham H. (2016). ggplot2-Elegant Graphics for Data Analysis (Cham, Switzerland: Springer International Publishing).

[B79] Wickham H. Averick M. Bryan J. Chang W. Mcgowan L. D. A. François R.. (2019). Welcome to the tidyverse. J. Open Source softw. 4, 1686. doi: 10.21105/joss.01686

[B80] Wijeyeweera R. Kleinberg I. (1989). Arginolytic and ureolytic activities of pure cultures of human oral bacteria and their effects on the pH response of salivary sediment and dental plaque in *vitro* . Arch. Oral. Biol. 34, 43–53. doi: 10.1016/0003-9969(89)90045-9 2675800

[B81] Yaling L. Tao H. Jingyi Z. Xuedong Z. (2006). Characterization of the Actinomyces naeslundii ureolysis and its role in bacterial aciduricity and capacity to modulate pH homeostasis. Microbiol. Res. 161, 304–310. doi: 10.1016/j.micres.2005.11.002 16412620

[B82] Yoon J. H. Yook J. I. Kim H. J. Cha I. H. Yang W. I. Kim J. (2003). Solitary plasmacytoma of the mandible in a renal transplant recipient. Int. J. Oral. Maxillofac. Surg. 32, 664–666. doi: 10.1054/ijom.2002.0416 14636623

