# Supplementary material for: Transplantation impacts on the oral microbiome of kidney recipients and donors
Source: Front Microbiomes. 2023 Nov 3;2:1258290. doi: 10.3389/frmbi.2023.1258290 (PMC12993617; doi:10.3389/frmbi.2023.1258290)
Supplement: Supplementary file 1 [file DataSheet_1.docx]

Supplementary Material

A longitudinal study of the oral microbiome of kidney transplant recipients and donors

Paul M. Campbell^1^, Thomas Willmott^1^, Gavin J. Humphreys^1^, Oana Piscoran^2^, Houda Chea^2^, Angela M. Summers^2^, Joanne E. Konkel^3^, Christopher G. Knight^4^, Titus Augustine^2^ and Andrew J McBain^1*^

*** Correspondence:**Andrew J. McBain
andrew.mcbain@manchester.ac.uk

# Supplementary Figures and Tables

**Supplementary Table 1.** Pairwise beta diversity values obtained following significant overall PERMANOVA results. Pairwise differences are considered significant at adjusted p-value (q value) <0.05.

|  |  | Unweighted | | | Weighted | | | Jaccard | | | Bray-Curtis | | |
| --- | --- | --- | --- | --- | --- | --- | --- | --- | --- | --- | --- | --- | --- |
| **Groups Compared** | **Sample size** | **pseudo-F** | **p-value** | **q-value** | **pseudo-F** | **p-value** | **q-value** | **pseudo-F** | **p-value** | **q-value** | **pseudo-F** | **p-value** | **q-value** |
| DA vs DB | 25 | 1.176 | 0.264 | 0.264 | 0.579 | 0.750 | 0.750 | 1.174 | 0.194 | 0.208 | 1.416 | 0.146 | 0.202 |
| DA vs DC | 22 | 1.870 | 0.018 | **0.032** | 1.500 | 0.166 | 0.313 | 1.574 | 0.014 | **0.021** | 1.375 | 0.148 | 0.202 |
| DA vs RA | 57 | 1.398 | 0.119 | 0.137 | 0.978 | 0.389 | 0.530 | 1.204 | 0.113 | 0.130 | 0.865 | 0.543 | 0.571 |
| DA vs RB | 56 | 1.878 | 0.019 | **0.032** | 1.454 | 0.188 | 0.313 | 1.913 | 0.001 | **0.003** | 2.257 | 0.008 | **0.017** |
| DA vs RC | 53 | 3.793 | 0.001 | **0.004** | 3.042 | 0.013 | **0.039** | 2.722 | 0.001 | **0.003** | 2.440 | 0.008 | **0.017** |
| DB vs DC | 23 | 2.198 | 0.005 | **0.013** | 0.947 | 0.432 | 0.540 | 1.337 | 0.062 | 0.078 | 0.885 | 0.571 | 0.571 |
| DB vs RA | 58 | 2.423 | 0.006 | **0.013** | 1.446 | 0.179 | 0.313 | 1.881 | 0.003 | **0.008** | 2.899 | 0.004 | **0.012** |
| DB vs RB | 57 | 1.196 | 0.220 | 0.236 | 0.689 | 0.625 | 0.670 | 1.062 | 0.289 | 0.289 | 0.953 | 0.490 | 0.565 |
| DB vs RC | 54 | 4.337 | 0.001 | **0.004** | 3.251 | 0.007 | **0.026** | 2.867 | 0.001 | **0.003** | 2.394 | 0.001 | **0.004** |
| DC vs RA | 55 | 1.392 | 0.113 | 0.137 | 2.549 | 0.043 | 0.108 | 1.476 | 0.009 | **0.015** | 1.744 | 0.051 | 0.096 |
| DC vs RB | 54 | 1.465 | 0.073 | 0.110 | 1.060 | 0.343 | 0.515 | 1.313 | 0.039 | 0.053 | 1.362 | 0.109 | 0.182 |
| DC vs RC | 51 | 1.462 | 0.106 | 0.137 | 0.877 | 0.492 | 0.568 | 1.491 | 0.008 | **0.015** | 1.151 | 0.296 | 0.370 |
| RA vs RB | 89 | 2.118 | 0.005 | **0.013** | 3.442 | 0.007 | **0.026** | 1.667 | 0.007 | **0.015** | 4.322 | 0.001 | **0.004** |
| RA vs RC | 86 | 3.368 | 0.001 | **0.004** | 10.706 | 0.001 | **0.008** | 2.506 | 0.001 | **0.003** | 4.687 | 0.001 | **0.004** |
| RB vs RC | 85 | 3.960 | 0.001 | **0.004** | 5.091 | 0.001 | **0.008** | 2.739 | 0.001 | **0.003** | 3.729 | 0.001 | **0.004** |

Supplementary Table 2. DESeq2 Results showing all ASVs with significant differential abundance in comparisons of kidney transplant donors and recipients at different timepoints (comparison indicated in bold, where “X vs Y” is indicated, positive log2Fold-Change means increase in X compared to Y) (p adj. <0.05). Columns include taxonomy at different levels, where assigned, (Order, Family, Genus and Species), the baseMean (average of normalized count values divided by size factors from all samples), the log2Fold-Change (effect size estimate), the lfcSE (standard error estimate of the log2Fold-change), the test statistic (stat), the unadjusted significance value (p-value) and the False Discovery Rate (FDR) adjusted p-value (padj).

| ASV ID (**Comparison**) | Order | Family | Genus | Species | baseMean | log2FoldChange | lfcSE | stat | pvalue | padj |
| --- | --- | --- | --- | --- | --- | --- | --- | --- | --- | --- |
| **Recipients B vs A** |  |  |  |  |  |  |  |  |  |  |
| ff5230bec494aa59294d8502293a6519 | Pasteurellales | Pasteurellaceae | Actinobacillus | parahaemolyticus | 28.382 | -26.311 | 1.871 | -14.062 | <0.001 | <0.001 |
| 88002ab8756b40a42e16717132e47a28 | Fusobacteriales | Fusobacteriaceae | Fusobacterium | NA | 8.526 | -7.498 | 2.358 | -3.180 | 0.001 | 0.045 |
| 1608689532ec031cfd9807c7dfe325a2 | Lactobacillales | Carnobacteriaceae | Granulicatella | NA | 36.304 | -6.556 | 1.210 | -5.418 | <0.001 | <0.001 |
| 9ca30664548a41cccc4964bf6be8cc9c | Pasteurellales | Pasteurellaceae | Actinobacillus | NA | 100.188 | -5.800 | 1.565 | -3.707 | <0.001 | 0.009 |
| ad4491df2aa2a9d107efeb75973869b9 | Pasteurellales | Pasteurellaceae | Haemophilus | NA | 110.048 | -5.649 | 1.109 | -5.091 | <0.001 | <0.001 |
| 1795839a8ad3b6217d0be534dc65d859 | Clostridiales | Veillonellaceae | Veillonella | NA | 20.035 | -4.166 | 1.184 | -3.520 | <0.001 | 0.016 |
| 5ab210b70bbb9d11943b629b9c1adea3 | Pasteurellales | Pasteurellaceae | Haemophilus | parainfluenzae | 969.028 | -1.627 | 0.438 | -3.714 | <0.001 | 0.009 |
| a2397abbce9d4a1bbf9d45e8c61edbf3 | Gemellales | Gemellaceae | NA | NA | 858.806 | -1.368 | 0.421 | -3.246 | 0.001 | 0.039 |
| b7af40a0cf39e917add7c0f1f6f1baf4 | Lactobacillales | Aerococcaceae | Abiotrophia | NA | 568.474 | 2.872 | 0.650 | 4.416 | <0.001 | 0.001 |
| 4a723d5beae43e83e6585cea8707c0c7 | Actinomycetales | Actinomycetaceae | Actinomyces | NA | 6.184 | 23.077 | 2.435 | 9.476 | <0.001 | <0.001 |
| **Recipients C vs A** |  |  |  |  |  |  |  |  |  |  |
| 9ca30664548a41cccc4964bf6be8cc9c | Pasteurellales | Pasteurellaceae | Actinobacillus | NA | 82.852 | -27.073 | 1.527 | -17.727 | <0.001 | <0.001 |
| 3fd3a4311764c58e881c0e17d53540d7 | Pasteurellales | Pasteurellaceae | Aggregatibacter | NA | 46.154 | -26.478 | 1.704 | -15.543 | <0.001 | <0.001 |
| 389763df5fa068c39f1819ff79710f16 | Clostridiales | Lachnospiraceae | NA | NA | 11.703 | -24.613 | 2.248 | -10.950 | <0.001 | <0.001 |
| ad4491df2aa2a9d107efeb75973869b9 | Pasteurellales | Pasteurellaceae | Haemophilus | NA | 89.802 | -9.378 | 1.037 | -9.042 | <0.001 | <0.001 |
| d84b9740ccb18376926052c85468984d | Flavobacteriales | Flavobacteriaceae | Capnocytophaga | NA | 7.794 | -5.490 | 1.635 | -3.358 | 0.001 | 0.025 |
| eff784a8bb5224c68442ad155be4fc32 | Pasteurellales | Pasteurellaceae | Aggregatibacter | segnis | 39.926 | -4.866 | 1.035 | -4.702 | <0.001 | <0.001 |
| 11dda1b32755394d15772f91c5d780da | CW040 | NA | NA | NA | 26.013 | -4.595 | 1.336 | -3.440 | 0.001 | 0.023 |
| 4a9f1c59cf525c06e83d681949bb611a | Bacteroidales | [Paraprevotellaceae] | [Prevotella] | NA | 153.404 | -4.089 | 1.084 | -3.773 | <0.001 | 0.008 |
| 81100c83423b1d27ddc85c1626790307 | Clostridiales | Peptococcaceae | Peptococcus | NA | 6.722 | -3.969 | 0.979 | -4.053 | <0.001 | 0.003 |
| fa3c31c44460408b8a4f40ec20ac7911 | Clostridiales | Peptostreptococcaceae | Peptostreptococcus | NA | 72.498 | -2.751 | 0.822 | -3.348 | 0.001 | 0.025 |
| 609b93fa4edf56f39d7a8c2d888b99b1 | Flavobacteriales | [Weeksellaceae] | NA | NA | 62.065 | -1.930 | 0.519 | -3.720 | <0.001 | 0.009 |
| ad4cd5d588129069899b6d0c45e64102 | Actinomycetales | Actinomycetaceae | Actinomyces | NA | 909.805 | 1.264 | 0.394 | 3.210 | 0.001 | 0.037 |
| a4fca56e6634cd05d3b526974413f50e | Actinomycetales | Actinomycetaceae | Actinomyces | NA | 498.917 | 1.979 | 0.587 | 3.374 | 0.001 | 0.025 |
| f8191449a0469e57a327f6d85a8a9b93 | Clostridiales | NA | NA | NA | 34.287 | 2.201 | 0.697 | 3.158 | 0.002 | 0.042 |
| 00334777da66484cc6efd2d99c4e9fa7 | Lactobacillales | Streptococcaceae | Streptococcus | NA | 606.087 | 2.215 | 0.586 | 3.779 | <0.001 | 0.008 |
| **Donors B vs A** |  |  |  |  |  |  |  |  |  |  |
| ff5230bec494aa59294d8502293a6519 | Pasteurellales | Pasteurellaceae | Actinobacillus | parahaemolyticus | 10.973 | -23.346 | 2.718 | -8.588 | <0.001 | <0.001 |
| **Donors C vs A** |  |  |  |  |  |  |  |  |  |  |
| 458bd373436f0d57f4bb41196e75d23d | Fusobacteriales | Fusobacteriaceae | Fusobacterium | NA | 21.766 | -23.349 | 2.651 | -8.807 | <0.001 | <0.001 |
| b60ac5372162b2441df47d3712540b3c | Pasteurellales | Pasteurellaceae | Aggregatibacter | NA | 15.894 | -22.930 | 2.864 | -8.008 | <0.001 | <0.001 |
| 1795839a8ad3b6217d0be534dc65d859 | Clostridiales | Veillonellaceae | Veillonella | NA | 48.043 | -8.645 | 1.836 | -4.708 | <0.001 | <0.001 |
| 11dda1b32755394d15772f91c5d780da | CW040 | NA | NA | NA | 21.128 | -7.460 | 2.068 | -3.607 | <0.001 | 0.041 |

Supplementary Table 3. Results of ALDEx2 Output for kidney transplant donors and recipients at different timepoints (comparison in bold, where “X vs Y” is indicated, positive diff.btw means increase in X compared to Y). Columns include taxonomy at different levels, where assigned, (Order, Family, Genus and Species) the median between (diff.btw) and within (diff.win) differences, the median effect size (effect), the expected values of *P* (we.ep) and associated Benjamini-Hochberg corrected false discovery rate values for Welch’s *t*-tests (we.eBH), and two columns contain the expected and Benjamini-Hochberg corrected false discovery rate values for Wilcoxon tests (wi.ep and wi.eBH).

| ASV ID | Order | Family | Genus | Species | diff.btw | diff.win | effect | overlap | we.ep | we.eBH | wi.ep | wi.eBH |
| --- | --- | --- | --- | --- | --- | --- | --- | --- | --- | --- | --- | --- |
| **Recipients B vs A** |  |  |  |  |  |  |  |  |  |  |  |  |
| ad4491df2aa2a9d107efeb75973869b9 | Pasteurellales | Pasteurellaceae | Haemophilus | NA | -6.597 | 6.705 | -0.868 | 0.204 | <0.001 | 0.002 | <0.001 | 0.001 |
| 1608689532ec031cfd9807c7dfe325a2 | Lactobacillales | Carnobacteriaceae | Granulicatella | NA | -4.783 | 6.280 | -0.675 | 0.249 | <0.001 | 0.021 | <0.001 | 0.025 |
| 9ca30664548a41cccc4964bf6be8cc9c | Pasteurellales | Pasteurellaceae | Actinobacillus | NA | -3.566 | 7.850 | -0.476 | 0.311 | 0.001 | 0.099 | 0.006 | 0.199 |
| a2397abbce9d4a1bbf9d45e8c61edbf3 | Gemellales | Gemellaceae | NA | NA | -2.514 | 2.775 | -0.768 | 0.165 | <0.001 | 0.003 | <0.001 | <0.001 |
| 5ab210b70bbb9d11943b629b9c1adea3 | Pasteurellales | Pasteurellaceae | Haemophilus | parainfluenzae | -1.960 | 3.281 | -0.548 | 0.191 | <0.001 | 0.054 | <0.001 | <0.001 |
| e922586081ebfd3c1131765980752500 | Lactobacillales | Streptococcaceae | Streptococcus | NA | -1.298 | 1.920 | -0.596 | 0.276 | <0.001 | 0.022 | <0.001 | 0.033 |
| **Recipients C vs A** |  |  |  |  |  |  |  |  |  |  |  |  |
| ad4491df2aa2a9d107efeb75973869b9 | Pasteurellales | Pasteurellaceae | Haemophilus | NA | -7.081 | 6.092 | -1.015 | 0.170 | <0.001 | <0.001 | <0.001 | <0.001 |
| eff784a8bb5224c68442ad155be4fc32 | Pasteurellales | Pasteurellaceae | Aggregatibacter | segnis | -6.111 | 6.657 | -0.819 | 0.205 | <0.001 | 0.004 | <0.001 | 0.002 |
| 5ab210b70bbb9d11943b629b9c1adea3 | Pasteurellales | Pasteurellaceae | Haemophilus | parainfluenzae | -4.773 | 5.476 | -0.830 | 0.160 | <0.001 | <0.001 | <0.001 | <0.001 |
| fa3c31c44460408b8a4f40ec20ac7911 | Clostridiales | Peptostreptococcaceae | Peptostreptococcus | NA | -4.764 | 6.542 | -0.706 | 0.224 | <0.001 | 0.011 | <0.001 | 0.002 |
| 9ca30664548a41cccc4964bf6be8cc9c | Pasteurellales | Pasteurellaceae | Actinobacillus | NA | -4.150 | 7.244 | -0.563 | 0.291 | <0.001 | 0.028 | 0.006 | 0.124 |
| 1608689532ec031cfd9807c7dfe325a2 | Lactobacillales | Carnobacteriaceae | Granulicatella | NA | -4.081 | 6.675 | -0.556 | 0.295 | 0.002 | 0.095 | 0.003 | 0.102 |
| 81100c83423b1d27ddc85c1626790307 | Clostridiales | Peptococcaceae | Peptococcus | NA | -3.812 | 5.223 | -0.656 | 0.236 | 0.001 | 0.040 | <0.001 | 0.013 |
| 64e1a83810f5e8b23458a6f4b5f7631a | Lactobacillales | Streptococcaceae | Streptococcus | NA | -2.905 | 6.051 | -0.436 | 0.308 | <0.001 | 0.014 | 0.002 | 0.126 |
| 609b93fa4edf56f39d7a8c2d888b99b1 | Flavobacteriales | [Weeksellaceae] | NA | NA | -2.669 | 3.940 | -0.607 | 0.207 | <0.001 | 0.005 | <0.001 | <0.001 |

## Supplementary Figures


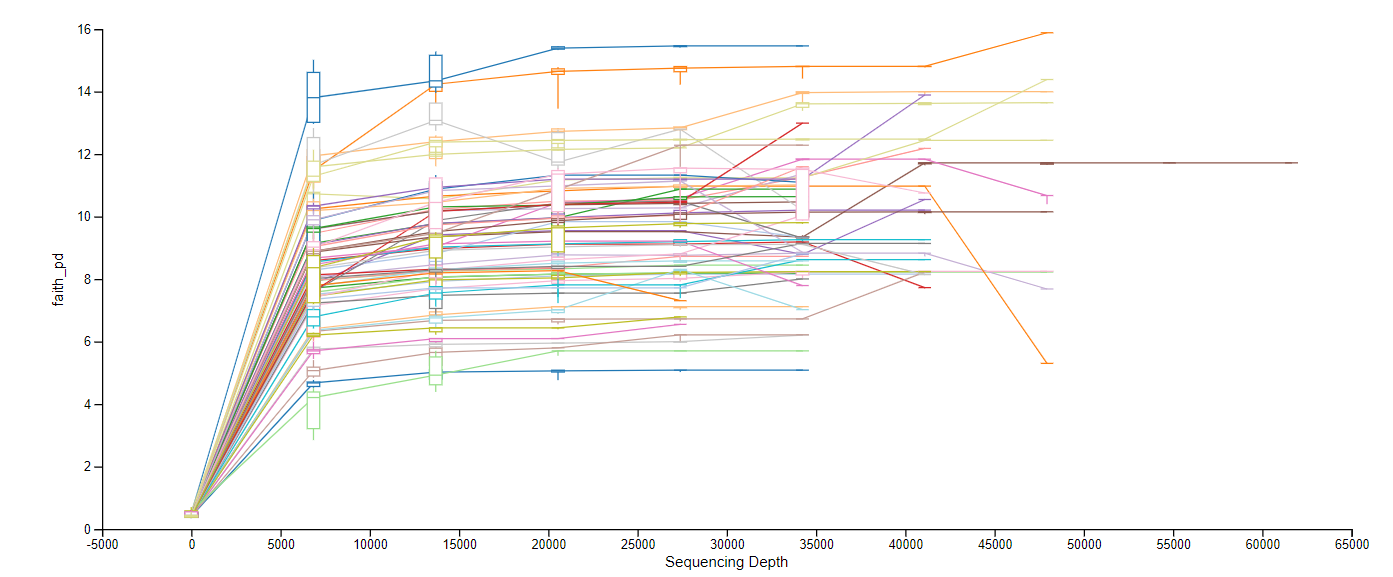


**Figure S1**. Alpha rarefraction plot (faith PD) showing sequencing depth for all samples.


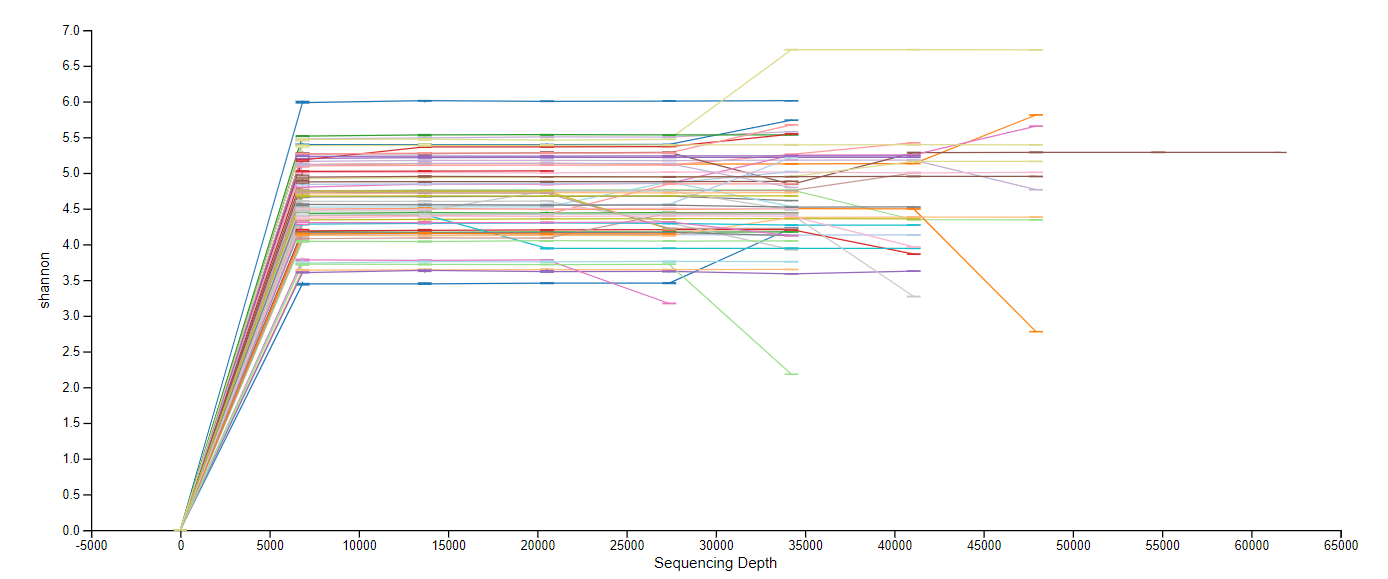


**Figure S2**. Alpha rarefraction curve (Shannon diversity index) showing sequencing depth for all samples.
